# Does gap-free intensity modulated chemoradiation therapy provide a greater clinical benefit than 3D conformal chemoradiation in patients with anal cancer?

**DOI:** 10.1186/1748-717X-7-201

**Published:** 2012-11-29

**Authors:** Claire Vautravers Dewas, Philippe Maingon, Cécile Dalban, Aurélie Petitfils, Karine Peignaux, Gilles Truc, Etienne Martin, Cédric Khoury, Sylvain Dewas, Gilles Créhange

**Affiliations:** 1 Department of Radiation Oncology, Anticancer center Georges François Leclerc, 1 rue du Professeur Marion, Dijon, 21000, France; 2 Department of Biostatistics, Anticancer center Georges François Leclerc, 1 rue du Professeur Marion, Dijon, 21000, France; 3 Departement of Radiation Oncology, Centre Oscar Lambret, Cedex 3 rue Frédéric combemale B.P, LILLE, 307 59020, France

**Keywords:** Anal canal cancer, Chemoradiation, IMRT, 3D-CRT, Toxicity

## Abstract

**Background:**

Chemoradiation is the standard treatment for anal cancer. 3D conformal radiotherapy (3D-CRT) is usually split in 2 sequences with a therapeutic break (gap) in between. Intensity-modulated radiation therapy (IMRT) makes it possible to reduce treatment time by abandoning this gap. The purpose of this study was to compare outcomes and toxicities in patients treated with either IMRT or 3D-CRT.

**Methods:**

Between 2004 and 2011, the data of 51 patients treated with exclusive radiotherapy with or without concomitant chemotherapy for non-metastatic anal carcinoma were retrospectively analyzed. Twenty-seven patients were treated with 3D-CRT and 24 patients with IMRT, with a median dose delivered to the tumor of 59.4Gy [30.6-66.6], whatever the radiotherapy technique (p= 0.99). The median follow-up was 40 months [26.4-51.6].

**Results:**

There was no difference between the two groups for response to treatment (p= 0.46). Two-year overall survival, locoregional relapse-free survival and colostomy-free survival rates were 88.5%, 63% and 60.3%, respectively for the IMRT group and 81%, 76.5% and 81.1% for the 3D-CRT group (all NS). Ten patients (37%) in 3D-CRT and 11 patients (45.8%) in IMRT (p= 0.524) had grade 3 acute toxicity. No grade 4 toxicity occurred.

**Conclusions:**

Our study suggests that further investigations concerning the use of IMRT to treat cancer of the anus are warranted. IMRT makes it possible to remove the gap, but with no impact on the prognosis. Nonetheless, a longer follow-up is essential to determine whether or not IMRT has an impact on late toxicity, local control and survival compared with conventional 3D-CRT.

## Background

Several randomized trials have shown that radiochemotherapy with 5-Fluorouracil (5FU) and Mitomycin-C (MMC) as the standard gave, at 4 years, an overall survival rate ranging from 60% to 72%, local control from 61% to 84% and colostomy-free survival of approximately 70% [1-3]. 3D conformal radiotherapy is usually delivered with a therapeutic break (gap) between two sequences. The EORTC (European Organization for Research and Treatment of Cancer) 22953 phase II trial showed that the duration of the gap between two sequences could be reduced from 6 to 2 weeks by decreasing the prophylactic dose delivered to the lymph nodes during the first sequence to 36 Gy [4]. The gap provides a number of advantages. It allows healthy tissues to regenerate and thus diminishes acute toxicity and non-planned treatment interruptions. Nonetheless, the gap could be detrimental for local control as it allows tumor cells to proliferate. A post hoc analysis of the RTOG (Radiation Therapy Oncology Group) 92-08 phase II trial retrospectively compared a cohort of patients treated with 3D-CRT with or without a gap [5]. At 8 years the rates of locoregional control (LRC), colostomy-free survival (CFS) and overall survival (OS) were better in the absence of a gap. In the literature, the impact of treatment duration on survival and LRC remains a matter of debate. Certain studies reported that prolonged treatment with more frequent treatment interruptions was associated with a poorer prognosis [5-7]. In contrast, other studies showed no link between the proportion of interruptions or total treatment time and poor control or lower survival [8-10].

The acute toxicity of radiochemotherapy delivering a dose around 55Gy is not negligible. The interest IMRT lies in the need to reduce the dose to healthy organs while maintaining the required dose to the target volume. IMRT may provide better protection of organs at risk (OAR). The advantages of IMRT for other pelvic sites are well known. There are, however, few data for IMRT in the treatment of the anal cancer [6,11-14]. Dosimetric studies that compared IMRT with 3D-CRT reported that IMRT provided better protection for healthy tissues, and several retrospective clinical studies reported that acute gastrointestinal and skin morbidity was lower than that in the RTOG 9811 trial [6,11-15].

We hypothesized that with IMRT it would be possible to permanently abandon the gap, maintain acceptable levels of toxicity and improve local control. In this attempt, we chose to abandon the gap rapidly after the implementation of IMRT in our institution. The controversial results of retrospective preliminary published studies prompted us to retrospectively review the outcomes and toxicities of anal cancer patients undergoing exclusive 3D-CRT or exclusive IMRT with and without a gap period at the Georges François Leclerc Cancer Center (Burgundy, France) over the last decade.

## Methods

### Patients

The data for all the 51 patients treated with radiotherapy with or without chemotherapy for non-metastatic anal carcinoma in the Georges François Leclerc Cancer Center (Burgundy, France) from 2004 to 2010 were analyzed retrospectively. All of the patients had a thorough examination before the treatment, including a clinical examination, a thoracic-abdominal-pelvic CT-scan and a biopsy. The characteristics of the patients and the tumors are reported in Table 1.

**Table 1:** Characteristics of patients and tumors according to the type of treatment (3D-CRT versus IMRT)

		**3D-CRT (n= 27)**	**IMRT (n= 24)**	**Total (n= 51)**	**p-value Chi2**
**Age**	Median	36.1 [40.7-92.2]	59.7 [49.8-88.1]	60.8 [40.7-92.2]	0.2575
**Sex**	Male	8 (29.6%)	8 (33.3%)	16 (31.4%)	0.776
	Female	19 (70.4%)	16 (66.7%)	35 (68.6%)	
**HIV**	No	8 (88.9%)	9 (100%)	17 (94.4%)	1
	Yes	1 (11.1%)		1 (5.6%)	
**WHO**	0	13 (54.2%)	18 (78.3%)	31 (66%)	0.233
	1	8 (33.3%)	4 (17.4%)	12 (25.5%)	
	2	3 (12.5%)	1 (4.4%)	4 (8.5%)	
**T-stage**	T1/T2/Tis	12 (44.4%)	11 (47.8%)	23 (46%)	
	T3/T4	15 (55.6%)	12 (52.2%)	27 (54%)	0.811
	Tx	0	1 (4.2%)	1 (2%)	
**N-stage**	N0	17 (65.4%)	10 (41.7%)	27 (54%)	0.093
	N1/N2/N3	9 (34.6%)	14 (58.3%)	23 (46%)	
	Nx	1 (3.7%)	0	1 (2%)	
**Histology**	Squamous cell	23 (85.2%)	22 (91.7%)	45 (88.2%)	0.671
	Others	4 (14.8%)	2 (8.3%)	6 (11.8%)	

*WHO*: World Health Organization, *HIV*: Human immunodeficiency virus.

### Radiochemotherapy

Before the treatment, all patients had a simulation CT-scan acquired in supine position. The first radiation sequence delivered 30 to 45Gy, with 2 to 4 beams in 3D-CRT and 4 to 9 beams in IMRT. The second sequence delivered a dose of up to 59.4Gy to a reduced volume including the tumor and the invaded lymph nodes, using 4 beams in 3D-CRT, and 4 to 9 beams in IMRT. In the case of a unique sequence, median dose delivered continuously and the technique used were similar to that described above for the second sequence. For all locally advanced tumors (T3, T4, and/or N+), the first target volume concerned the tumor and the full pelvis, including mesorectal nodes, bilateral inguinal nodes, internal and external iliac lymph nodes. For T1-T2 and N0 tumors, the first target volume concerned the tumor and a smaller pelvic field which ruled out the inguinal nodes. The delineation of the volumes and the doses delivered during the first and the second sequence of the radiotherapy were similar whatever the technique used, 3D-CRT or IMRT. The second target volume concerned the tumor and the initially-invaded lymph nodes. In order to take into account interfraction and set-up uncertainties, the margin applied around both target volumes was 10 mm.

After 2008, all patients with anal carcinoma were treated with IMRT, whatever the target volume.

The treatment was planned on Eclipse software (Varian Medical Systems, Palto Alto, CA). For the IMRT, the priority was maximal coverage of the PTV, while the secondary objective was to spare OAR (bladder, small bowel (SB), femoral heads). Organs at risk were outlined identically over time using the same policy in our institution. Genitals were not considered as a separate structure and no dose limits were applied. The SB was defined as the abdominal cavity, thus including both the small and large bowels and the visceral fat surrounding the loops. The objectives of IMRT for the PTV were that 100% should receive at least 95% of the prescribed dose, 0% more than 107%, 50% more than the prescribed dose and 50% less than the prescribed dose. The classical dose-volume constraints for the IMRT planning were: 30% of the SB should receive <30 Gy, 50% of the bladder should receive <50 Gy and the mean dose to the FH should be <45 Gy. The IMRT plan was optimized to minimize the proportion of both the PTV receiving <95% and >107% of the prescribed dose. The isodose of the prescription was 100%. Generally, the dose was normalized to the mean dose planned in the target volume.

**Figure 1:**
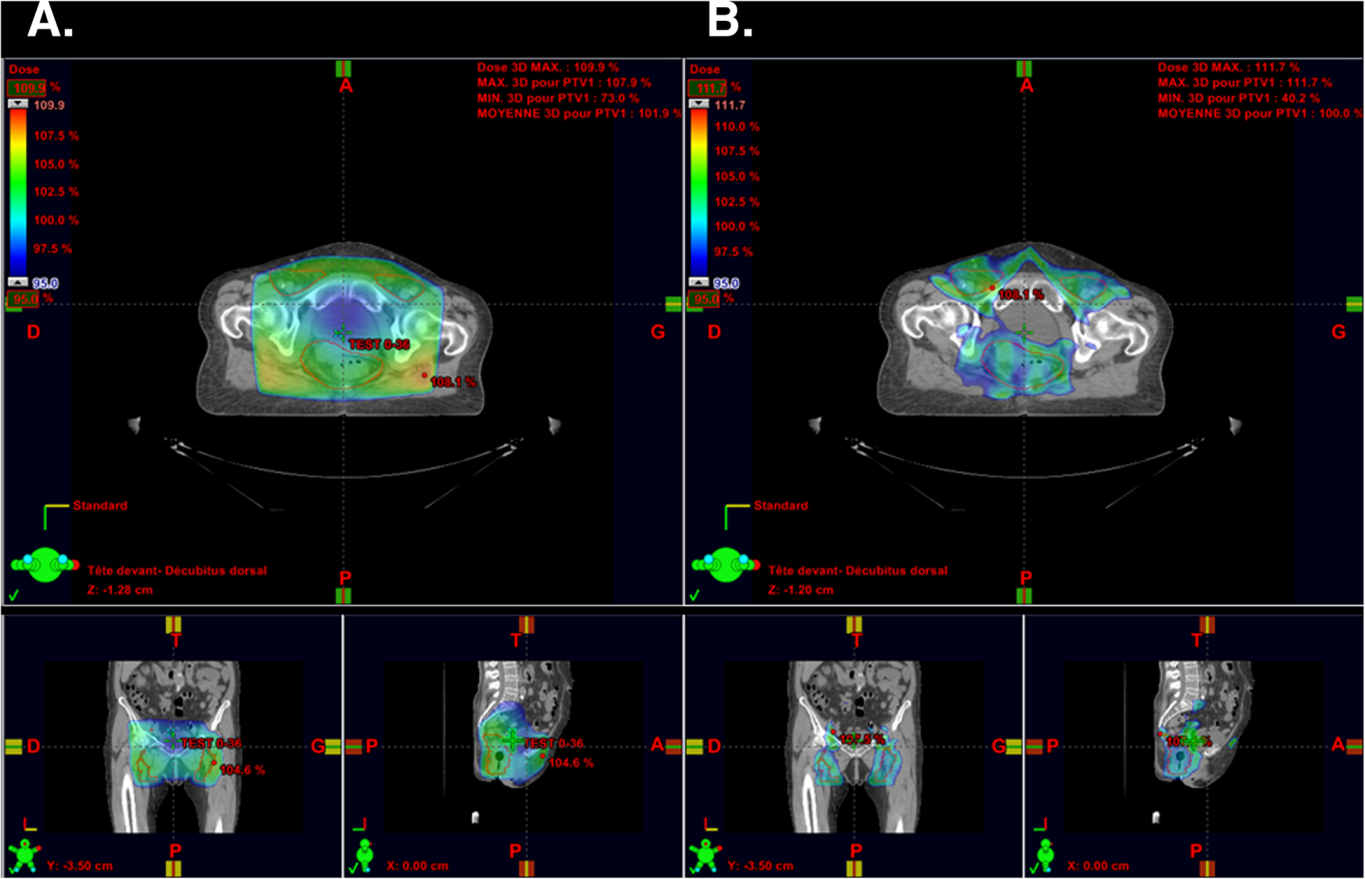
**Comparison of dose distribution on planning CT with 3D-CRT and IMRT for a same patient with a locally advanced T3N+ anal cancer.** Legend: The isodose distribution shows up in colorwash with the 95% isodose line selected Left column (**A**.) axial CT slice using 3D-CRT technique with small coronal and sagittal views above; Right column (**B**.) axial CT slice using IMRT technique. with small coronal and sagittal views above.

**Figure 2:**
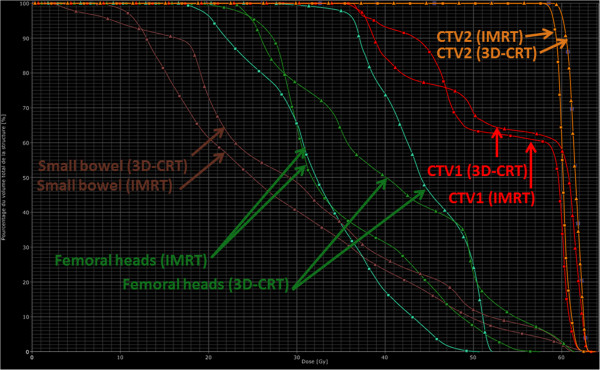
Dose Volume Histogram (DVH) comparison of 3D-CRT vs. IMRT for the same anal cancer patient.

The schemes of concomitant chemotherapy were those of the EORTC 22953 (5FU+MMC) or the EORTC 22011 study (CDDP+MMC) [16]. Figure 1 represents the pelvic dose distribution using 3D-CRT vs. IMRT for the same anal cancer patient and Figure 2 shows the DVH comparison.

### Dosimetric analysis

This analysis was done on all the 38 patients who were treated on the full pelvis, the 13 other patients were systematically excluded. The dosimetric data were retrospectively collected. For the FH, the mean dose was recorded. For the SB, the mean dose (Dmean), the maximal dose (Dmax), as well as the volume receiving 30 Gy (V30), V40, V50 and V60 were studied. For the bladder, Dmean, Dmax and the minimal dose (Dmin) as well as the dose delivered to 95% of the bladder (D95) were analyzed. The Dmax, Dmin, Dmean and D95 to PTV1 and PTV2 were analyzed.

### Gap

For locally advanced tumors treated with 3D conformal concomitant chemoradiotherapy, a 2-week gap was planned according to the EORTC 22953 trial. In 2008, with the introduction of IMRT, the gap was quickly abandoned in routine practice.

### Toxicity

During radiotherapy, patients had a consultation at least once a week, during which toxicity were prospectively recorded and graded according to the CTCAE v.3.0. Toxicity that became apparent 6 months after the treatment was considered late.

### Evaluation of the response and follow-up

A complete response (CR) was defined as the absence of any residual tumor, partial response (PR) as the persistence of a lesion after a response of more than 30%, and stable disease (SD) as a response to the treatment of less than 30%. After the treatment, the patients were seen every 3 months for one year, and then once every 4 months for 3 years. In cases of remission, the patients were seen every 6 months thereafter. A digital rectal examination and anuscopy were performed. Biopsy or imaging examinations were conducted only when there were signs. Local recurrence was defined as the appearance of a clinical macroscopic lesion, whether or not it was proven histologically from a biopsy, at the site of the initial disease.

### Statistics

The median follow-up was calculated using the Reverse Kaplan-Meier method. The Kaplan Meier method was used to determine OS, Loco-regional Relapse-Free Survival (LRFS), Disease-Free Survival (DFS) and CFS. These survivals were calculated from the date of diagnosis to the event, to death or to the date of the last news. The survival curves were compared using the Log Rank test. Uni- and multivariate analyses were done using the Cox model. The statistical analysis was done using STATA V11 (STATA Corp, College Station, TX) software.

## Results

Of the 51 patients, 27 were treated with 3D-CRT and 24 with IMRT. Analysis of the 2 groups revealed no significant differences for the characteristics of either the patients or the tumors (Table 1).

### Radiotherapy

In 38 patients (74.5%) the full pelvis was irradiated with 2 to 4 fields in 3D-CRT, and 4 to 9 fields in IMRT. The repartition of those patients between the 2 techniques is well balanced, 18 treated with 3D-CRT and 20 with IMRT (p=0.173). Twenty-two had a gap during the treatment, and 16 did not have a programmed break.

The median dose delivered to the CTV1 was 36Gy [30.0-45.0], with no difference for the technique ([30.6-45] for IMRT and [30-45] for 3D-CRT). The median dose delivered to the CTV2 was 59.4Gy [30.6-66.6], with no difference for the technique ([30.6-65] for IMRT and [32.4-66.6] for 3D-CRT). Forty-nine patients (96%) received the totality of the initially-planned treatment.

### Chemotherapy

Forty-one patients (80.4%) had concomitant chemotherapy: capecitabine alone (1), MMC combined with 5FU or capecitabine (36), MMC and CDDP (4). Nineteen were treated with 3D-CRT and 22 with IMRT. Twenty-two had a gap during the treatment, and 19 did not have a programmed break.

For the 10 patients not treated with chemotherapy, the reason was the tumor stage (T1-T2 and N0), the age (over 80 years old) or a refusal. The patients receiving chemotherapy or not were equally divided between 3D-CRT versus IMRT, p= 0.081.

### Duration of treatment and interruptions

Median duration of the treatment was 56 days [22-103] (59 versus 47 days with 3D-CRT and IMRT respectively, p= 0.0007). A gap was planned in 29 patients (57%), 23 with 3D-CRT and 6 with IMRT (p< 0.0001). Treatment was stopped for toxicity in 9 patients (17.6%), 4 with 3D-CRT and 5 with IMRT (p= 0.48).

### Toxicity

Acute and late toxicity is presented in Table 2.

**Table 2:** Acute and late toxicity

		**G1/G2**				**G3/G4**			
		**3D-CRT n=27**	**IMRT n=24**	**Total n=51**	**P value**	**3D-CRT n=27**	**IMRT n=24**	**Total n=51**	**P value**
**Acute**	**Perineal skin**	13 (48.1%)	13 (54.2%)	26 (51%)	0.756	9 (33.3%)	9 (37.5%)	18 (35.3%)	0.756
	**Diarrhea**	12 (0.44%)	11 (45.8%)	23 (45.2%)	1	1 (3.7%)	1 (4.2%)	2 (3.9%)	1
	**Pain**	10 (37%)	15 (62.5%)	25 (49%)	0.331	1 (3.7%)	3 (12.5%)	4 (7.8%)	0.331
	**Neutropenia**	1 (0.04%)	0	1 (2%)	1	1 (3.7%)	1 (4.2%)	2 (3.9%)	1
**Late**	**Rectal bleeding**	6 (22.2%)	6 (0.25%)	12 (24.5%)	1	1 (3.9%)	0	1	1
	**Impotence**	0	0	0	1	1 (12.5%)	0	1	1

Two patients stopped definitively the treatment around 30Gy because of toxicity (rectovaginal fistula and G3 perineal skin toxicity). Ten patients (37%) in 3D-CRT and 11 (45.8%) in IMRT, (p= 0.524) presented G3 acute hematological and non-hematological toxicity. There were no cases of G4 toxicity. One patient in the 3D-CRT group and 5 in the IMRT group developed G3 acute digestive toxicity (p= 0.088). Concerning the G3 perineal skin toxicity, there were 9 cases in the 3D-CRT group and 9 in the IMRT group. Two patients in the 3D-CRT group and none in the IMRT group had G3 late toxicity (p= 0.491).

### Clinical response

Forty-nine patients (96.1%) were examined during the 3 months following the end of the treatment. Thirty-nine patients (79.6%) had CR, 9 (18.3%) PR and 1 (2%) disease progression. There was no difference between the 2 groups for response to treatment (p= 0.46).

### Follow-up and outcomes

The median follow-up for the whole cohort was 40 months [26.4-51.6], 60 months [45.6-69.6] for 3D-CRT and 23 months [15.6-38.4] for IMRT. At the time of the analysis, 34 patients (66.6%) were still alive: 33 were alive without disease, and 1 in the IMRT group presented a locoregional and metastatic recurrence.

Median OS was 5.1 years. The OS rate at 2 years was 84.9% (81.1% 3D-CRT versus 88.5% IMRT, p= 0.58). LRFS rate at 2 years was 71.5% (76.5% 3D-CRT versus 63% IMRT, p= 0.43). CFS rate at 2 years was 73.6% (81.1% 3D-CRT versus 60.3% IMRT, p= 0.12).

Seven patients (13.7%) developed local recurrence, 4 with IMRT and 3 with 3D-CRT. Three patients underwent abdominoperineal resection (APR) and one patient had R0 conservative surgery. Four patients (7.8%) developed recurrence in the lymph nodes, 3 with IMRT (one of these had associated local recurrence) and 1 with 3D-CRT.

### Dosimetric comparison of 3D-CRT and IMRT

The results of the analysis for PTV1 and 2 as well as for the organs at risk, depending on the radiotherapy technique used (3D-CRT versus IMRT), are presented in Table 3.

**Table 3:** Dosimetric analysis in the group of patients with pelvic radiotherapy (n= 38)

	**3D-CRT (n= 18) Mean value**	**IMRT (n= 20) Mean value**	**P value**
**Total dose**	58.3 Gy	58.9 Gy	0.366
**Urinary bladder**			
**D mean**	44.8 Gy	34.5 Gy	0.008
**Dmax**	59.8 Gy	55.4 Gy	0.363
**Dmin**	32.5 Gy	19.0 Gy	0.034
**D95**	34.1 Gy	22.8 Gy	0.061
**Right femoral**			
**head Dmean**	29.9 Gy	26.9 Gy	0.582
**Left femoral head**			
**Dmean**	32.8 Gy	27.1 Gy	0.133
**Small bowel**			
**Dmean**	13.4 Gy	17.8%	0.632
**Dmax**	47.8 Gy	46.3%	1
**V30**	16%	22.7%	0.719
**V40**	0%	8.5%	0.195
**V50**	0%	2.9%	0.273
**V60**	0%	0.5%	0.484
**PTV1**			
**Dmax**	62.1 Gy	62.3 Gy	0.473
**Dmin**	26.6 Gy	27.2 Gy	0.702
**Dmean**	51.1 Gy	50.3 Gy	0.962
**D95**	37.2 Gy	37.4 Gy	0.702
**PTV2**			
**Dmax**	62.2 Gy	62.3 Gy	0.426
**Dmin**	33.9 Gy	44.8 Gy	0.408
**Dmean**	59.9 Gy	58.8 Gy	0.655
**D95**	57.8 Gy	57.6 Gy	0.014

V30: volume of the considered organ receiving 30Gy or less (in %); V40: volume of the considered organ receiving 40Gy or less (in %); V50: volume of the considered organ receiving 50Gy or less (in %); V60: volume of the considered organ receiving 60Gy or less (in %); Dmax: maximal dose; Dmin: minimal dose; Dmean: mean dose; D95: dose delivered to 95% of the target.

3D-CRT was compared with IMRT for the whole population as well as for patients with radiation to the pelvis. For the FH and the SB, there was no significant difference between the groups, even for the pelvis subgroups. For the bladder, there was no difference between the two groups for the whole population. However, for the sub-group with treatment to the pelvis, the mean dose delivered to the bladder was 44.8Gy in 3D-CRT and 34.5Gy in IMRT (p< 0.007).

For PTV1 and 2, there was no significant difference between the 2 groups for the Dmax, Dmin, Dmean, and D95.

### Predictive factors for local control, and overall and colostomy-free survival

The factors tested in univariate analysis are reported in Table 4. In multivariate analysis, no factor had a significant impact on OS and DFS. With regard to LRFS, only N stage was statistically associated in multivariate analysis (p= 0.035). In multivariate analysis, only the WHO (World Health Organization) score had a significant impact on CFS (p= 0.032).

**Table 4:** Predictors of local control, overall and colostomy-free survival in univariate analysis (n= 51)

	**Overall survival**			**Locoregional relapse-free survival**			**Colostomy-free survival**		
	**HR**	**CI 95%**	**p-value**	**HR**	**CI 95%**	**p-value**	**HR**	**CI 95%**	**p-value**
**WHO**									
**0**	**1**	**[0.99-7.17]**	**0.053**	**1**	**[0.83-5.64]**	**0.115**	**1**	**[1.3-8.07]**	**0.012**
**1/2**	**2.66**			**2.16**			**3.24**		
**T Stage**									
**T1/T2/in situ**	**1**	**[0.45-3.42]**	**0.684**	**1**	**[0.38-2.64]**	**1**	**1**	**[0.44-2.73]**	**0.846**
**T3/T4**	**1.23**			**1**			**1.1**		
**N Stage**									
**N0**	**1**	**[0.95-7.5]**	**0.062**	**1**	**[0.97-7.46]**	**0.057**	**1**	**[1.04-6.91]**	**0.042**
**N1/N2/N3**	**2.67**			**2.69**			**2.68**		
**Histology**									
**Squamous cell cancer other**	**1 0.86**	**[0.2-3.82]**	**0.846**	**1 0.83**	**[0.19-3.65]**	**0.809**	**1 0.68**	**[0.16-2.97]**	**0.612**
**Treatment duration**									
**<56 days**	**1**	**[0.26-1.96]**	**0.511**	**1**	**[0.23-1.60]**	**0.317**	**1**	**[0.23-1.49]**	**0.262**
**>56 days**	**0.71**			**0.61**			**0.59**		
**Total dose**									
**<50 Gy**	**1**	**[0.11-6.47]**	**0.874**	**1**	**[0.14-8.09]**	**0.951**	**1**	**[0.04-0.88]**	**0.033**
**>50 Gy**	**0.85**			**1.07**			**0.19**		
**Technique**									
**3D-CRT**	**1**	**[0.46-3.94]**	**0.580**	**1**	**[0.55-3.98]**	**0.431**	**1**	**[0.81-5.66]**	**0.125**
**IMRT**	**1.35**			**1.48**			**2.14**		
**Treatment breaks for toxicity**									
**Yes**	**1**	**[0.23-4.46]**	**0.996**	**1**	**[0.37-4.56]**	**0.680**	**1**	**[0.16-2.96]**	**0.607**
**No**	**1**			**1.3**			**0.68**		
**Planned gap**									
**Yes**	**1**	**[0.29-3.17]**	**0.943**	**1**	**[0.3-2.4]**	**0.765**	**1**	**[0.37-3.17]**	**0.882**
**No**	**0.96**			**0.85**			**1.08**		
**Pelvic radiotherapy**									
**Yes**	**1**	**[0.34-3.33]**	**0.905**	**1**	**[0.44-5.35]**	**0.501**	**1**	**[0.45-4.1]**	**0.587**
**No**	**1.07**			**1.54**			**1.36**		

### Predictive factors for G3 or G4 acute toxicity

No factors were associated with the appearance of G3+ toxicity, neither in uni- nor in multivariate analysis. In particular, the radiotherapy technique had no impact.

## Discussion

Studies involving series of patients treated with radiochemotherapy, associating 2D radiotherapy with 5FU and MMC, reported rates of LRC at 4 years (4y) ranging from 61% to 84% [1-3,15]. Studies that used IMRT reported rates of LRC from 83.9% to 91.9% (3y) [6,12-14,17-20]. In the study of Bazan *et al.* LRC with IMRT was significantly greater than that with 3D non conformal RT (3y, 92% versus 57%, p< 0.01) [6]. In our study, LRC at 2 years was 65.8% in IMRT versus 88% in 3D-CRT (p= 0.21). With IMRT, we found 12.5% of lymph nodes recurrences in a population of whom 58.3% were node positive (N+). This finding is in keeping with published data for IMRT [12,20]. We found in multivariate analyses, that the technique used was not a predictor of LRFS. Nevertheless, it remains difficult to compare IMRT results across institutions because of technical differences and learning curves needed with IMRT. The quality assurance of radiotherapy is often lacking in such studies and should be mandatory in the future when delivering IMRT.

OS from 58% to 75% (5y) has been reported in series of patients undergoing 2D-based radiochemotherapy [1-3,15]. In the majority of studies on IMRT, OS ranged from 63% to 94% (2y). Bazan *et al.* reported significantly greater OS in patients treated with IMRT than in those treated with 3D non-conformal RT (3y, 88% versus 52%, p< 0.01) [6]. In our study, 2y-OS was 81.1% in the 3D-CRT and 88.5% in the IMRT group (p= 0.58).

CFS of around 70% at 4y with 2D radiochemotherapy has been reported in the literature [1-3,15]. Data for patients treated with IMRT showed 3y-CFS from 82% to 91%. In our study, 2y-CFS was 81.1% in the 3D-CRT and 60.3% in the IMRT group (p= 0.12). Five patients, all of whom were in the IMRT group, had a colostomy for rectovaginal fistula (1), local recurrence (2), and for comfort in a context of incontinence (2).

Several studies explored the benefits of IMRT with regard to reducing adverse effects (Table 5). For Bazan *et al.* G3+ non-hematological toxicity were significantly more frequent in the 3D-RT group (65% versus 21%, p= 0.003) [6]. In multivariate analysis, the risk of developing G3+ toxicity was significantly lower with IMRT than with 3D-RT. The incidence of G3+ acute skin toxicity with 2D-RT was 48% in the 5FU+MMC arm of the RTOG 98-11 trial, and 17% in the UKCCCR trial [1,15]. Studies that used IMRT reported skin toxicity ranging from 0 to 38% [6,12-14,17,20]. G3 acute toxicity was significantly less frequent with IMRT than with 3D-RT (21% versus 41%) [6]. In our cohort, we recorded 35.3% of G3 skin toxicity, and no G4 toxicity (33.3% 3D-CRT versus 37.5% IMRT, p= 0.756). With 2D-radiochemotherapy, acute G3+ gastrointestinal toxicity was 35% for Ajani *et al.* and 5% in the UKCCCR trial [1,15]. With IMRT the rate of G3+ acute digestive toxicity ranged from 0 to 66%. Bazan *et al.* reported 29% and 7% of G3+ acute digestive toxicity in 3D-RT compared with IMRT [6]. We found 6 (11.8%) G3 acute digestive toxicity (including anitis and diarrhea) in the 51 patients, 5/24 with IMRT (20.8%) and 1/27 (3.7%) with 3D-CRT (p= 0.088), and no G4 toxicity. In contrast to published studies that compared 3D non conformal RT with IMRT, 2 AP-PA beams were used most often. We believe our 3D technique was more conformal by delivering 2 to 4 fields. This difference in dose distribution may explain the discordance between our series and the literature concerning the low toxicity in 3D-CRT group.

**Table 5:** Grade 3+ acute toxicity and locoregional control: review of the literature for IMRT

	**Number of patients**	**Follow-up (months)**	**Locoregional control (%)**	**Treatment breaks (%)**	**Breaks (days)**	**Rates of grade 3+ GI toxicity (%)**	**Rates of grade 3+ skin toxicity (%)**	**Rate of grade 3 and 4 haematological toxicity (%)**	**Scale**
**Pepek*et al.***[13]	47	Median 14	90 (2-y)	18 (AT)	Median 5 (2-7)	10	0	24	CTC V3
**Bazan*et al.***[6]	46	Median							CTC V3
	17 (3D)	3D : 26	3D : 56.7 (3-y)	3D : 88 (AT)	3D : 12	3D : 29	3D : 41	3D : 29	
	29(IMRT	IMRT: 32	IMRT : 91.9 (3-y) (P<0.01)	IMRT : 34.5 (90% AT, 10% NC) (P=0.001)	IMRT : 1.5 (P<0.0001)	IMRT: 7	IMRT: 21	IMRT : 21	
**Salama*et al.***[14]	53	Median	83.9 (1.5-y)	41.5	Median	15	38	59	CTC V3
		14.5		(AT)	4 (2-14)				
**RTOG 0529*.***[19]	43	24	95 (2-y)	40	Median	7	10	61	CTC V3
				(AT+NC) 35 (toxicity)	2 (2-24)				
**Kachnic*et al.***[20]	52	23.2	80 (2-y)	NR	NR	22	20	NR	CTC V3
**Milano*et al.***[12]	17	20.3	82 (2-y)	24 (AT)	NR	0	0	38	RTOG
**Hodges*et al.***[17]	6	25	100 (3-y)	50	1- 3	67	0		CTC V3
**Vautravers-Dewas*et al.***	51	Median		17.6	Median	3D : 3.7	35.3	4	CTC V3
		3D : 60	88 (2-y)	(AT)	15 (1-43)	IMRT : 20.8			
		IMRT : 23	65.8 (2-y)			(P=0.088)			

*GI*: gastrointestinal; *AT*: acute toxicity; *NC*: not compliant; *CTC*: Common Toxicity Criteria; *NR* : not reported; *MMC*: Mitomycin C.

Retrospective studies have confirmed the need for non-programmed treatment breaks notably because of skin or digestive reactions. Ajani *et al.* reported a rate of breaks of 61% with 2D-chemoradiation [15]. With IMRT, the number of breaks for toxicity ranged from 18% to 50%. For Bazan *et al.* IMRT made it possible to significantly reduce the median duration of the treatment (57 versus 40 days, p< 0.0001) [6]. Breaks for toxicity were significantly more frequent with 3D-RT than with IMRT (88% versus 34.5%, p= 0.001). In our study, we found 11.1% and 20.8% of breaks for toxicity for 3D-CRT and IMRT, respectively (p= 0.48). Our rate of breaks with IMRT was comparable to Bazan *et al.* (20.8% versus 24.1%) [6]. However, we found far fewer breaks with 3D-CRT (11.1% versus 88%), which very probably explains the discordance of the results with regard to the impact of the technique used.

With IMRT it is possible to reduce total treatment time: median treatment time was 49 and 42 days in the RTOG 98-11 trial (3D) and the 0529 trial (IMRT), respectively [18]. Certain studies reported that prolonged treatment and more frequent treatment breaks were associated with a poorer prognosis [5,7]. In the series by Bazan *et al.* the patients who did not interrupt treatment had better OS, LRC and PFS at 3 years than those who had breaks (respectively 90% versus 45% p= 0.03, 95% versus 67% p= 0.02, 89% versus 63% p= 0.04) [6]. In contrast, other studies showed no association between the percentage of breaks and total treatment time, and poor control or diminished survival [9,10]. Although Ben Joseph *et al.* found in a post hoc analysis of RTOG trials that overall treatment time was a significant predictor for local failure (but not overall survival), radiation dose, radiation duration and radiation intensity showed no correlation with colostomy failure or local failure rates in multivariate analysis [8]. Because patients in the experimental arm of RTOG 98-11 had significantly longer overall treatment time (4 courses of neoadjuvant 5-FU+Cisplatin), an additional analysis considering only patients treated with concomitant 5-FU + MMC but without neoadjuvant chemotherapy (i.e. mean overall treatment time of 45 days) showed no correlation but a trend (p= 0.078) between the duration of radiotherapy and the colostomy failure rate. In our study, we found no significant association between numbers of breaks, duration of the treatment, existence of a planned gap and prognosis for either LRC or survival.

Nonetheless, our analysis does include a number of limitations linked to the retrospective nature of the data collection, as a result of which the doses of radiation and the chemotherapy protocols were heterogeneous. Even though our median follow-up in the group of patients treated with IMRT was short, our results are comparable to those in series with a similar follow-up [13,14]. Our results suggest that the clinical benefit of IMRT is limited to the reduction in treatment time, and they must be considered with caution together with the preliminary data already published. We are now awaiting the results of the RTOG 0529 phase II trial, with a longer follow-up, to provide a definitive answer concerning the benefits of IMRT. A phase III trial to compare IMRT with 3D-CRT is required given the different results in the literature to conclusively address this issue. Quality of life using the two protocols also needs to be evaluated.

## Conclusion

Our study, even though it suffers from the classical limitations of a retrospective analysis, suggests that further investigations concerning the use of IMRT as a standard to treat anal cancer are necessary. IMRT makes it possible to reduce treatment time, notably by abandoning the gap, but with no impact on the prognosis. Nonetheless, a longer follow-up is essential to determine whether or not IMRT has an impact on late toxicity, local control and survival compared with conventional 3D-CRT.

## Abbreviation

5-FU: 5-Fluoro-Uracile; MMC: Mitomycin C; EORTC: European Organisation for Research and Treatment of Cancer; RTOG: Radiation Therapy Oncology Group; 3D-CRT 3: Dimensional Conformal Radiation Therapy; LRC: Locoregional Control; IMRT: Intensity Modulated Radiation Therapy; CFS: Colostomy-Free Survival; OS: Overall Survival; OAR: Organs At Risk; CDDP: Cisplatin; CTCAE: Common Toxicity Criteria Adverse Events; SD: Standard Deviation; CR: Complete Response; PR: Partial Response; LRFS: Locoregional Relapse-Free Survival; DFS: Disease-Free Survival; CTV: Clinical Target Volume; APR: Abdomino-Perineal Resection; WHO: World Health Organization; UKCCCR: United Kingdom Co-ordinating Committee on Cancer Research.

## Competing interests

The authors declare that they have no competing interests.

## Authors’ contributions

Conception and design: CVD, PM, GC. Acquisition of Data: CVD, EM, KP, GT, AP, CK. Analysis and interpretation of data: CVD, CD, SD, AP, PM, GC. Drafting the manuscript or revising it critically for important intellectual content: CVD, AP, CD, PM, GC. Final approval of the version to be published: CVD, CD, SD, EM, KP, GT, CK, AP, PM, GC. All authors read and approved the final manuscript.
